# Horizon 2020 in Diabetic Kidney Disease: The Clinical Trial Pipeline for Add-On Therapies on Top of Renin Angiotensin System Blockade

**DOI:** 10.3390/jcm4061325

**Published:** 2015-06-18

**Authors:** Maria Vanessa Perez-Gomez, Maria Dolores Sanchez-Niño, Ana Belen Sanz, Catalina Martín-Cleary, Marta Ruiz-Ortega, Jesus Egido, Juan F. Navarro-González, Alberto Ortiz, Beatriz Fernandez-Fernandez

**Affiliations:** 1 Division of Nephrology and Hypertension and FRIAT, IIS-Fundacion Jimenez Diaz, School of Medicine, UAM, Av Reyes Catolicos 2, 28040 Madrid, Spain; E-Mails: mvanessa@fjd.es (M.V.P.-G.); mdsanchez@fjd.es (M.D.S.-N.); asanz@fjd.es (A.B.S.); cmartinc@fjd.es (C.M.-C.); mruizo@idcsalud.es (M.R.-O.); jegido@fjd.es (J.E.); aortiz@fjd.es (A.O.); 2 REDINREN, Av Reyes Catolicos 2, 28040 Madrid, Spain; 3 Nephrology Service and Research Unit, University Hospital Nuestra Señora de Candelaria, Carretera del Rosario, 145, 38010 Santa Cruz de Tenerife, Spain; E-Mail: jnavgon@gobiernodecanarias.org

**Keywords:** chronic kidney disease, diabetes, diabetic kidney disease, inflammation, interleukin-1-beta, treatment

## Abstract

Diabetic kidney disease is the most frequent cause of end-stage renal disease. This implies failure of current therapeutic approaches based on renin-angiotensin system (RAS) blockade. Recent phase 3 clinical trials of paricalcitol in early diabetic kidney disease and bardoxolone methyl in advanced diabetic kidney disease failed to meet the primary endpoint or terminated on safety concerns, respectively. However, various novel strategies are undergoing phase 2 and 3 randomized controlled trials targeting inflammation, fibrosis and signaling pathways. Among agents currently undergoing trials that may modify the clinical practice on top of RAS blockade in a 5-year horizon, anti-inflammatory agents currently hold the most promise while anti-fibrotic agents have so far disappointed. Pentoxifylline, an anti-inflammatory agent already in clinical use, was recently reported to delay estimated glomerular filtration rate (eGFR) loss in chronic kidney disease (CKD) stage 3–4 diabetic kidney disease when associated with RAS blockade and promising phase 2 data are available for the pentoxifylline derivative CTP-499. Among agents targeting chemokines or chemokine receptors, the oral small molecule C-C chemokine receptor type 2 (CCR2) inhibitor CCX140 decreased albuminuria and eGFR loss in phase 2 trials. A dose-finding trial of the anti-IL-1β antibody gevokizumab in diabetic kidney disease will start in 2015. However, clinical development is most advanced for the endothelin receptor A blocker atrasentan, which is undergoing a phase 3 trial with a primary outcome of preserving eGFR. The potential for success of these approaches and other pipeline agents is discussed in detail.

## 1. Introduction

**Figure 1:**
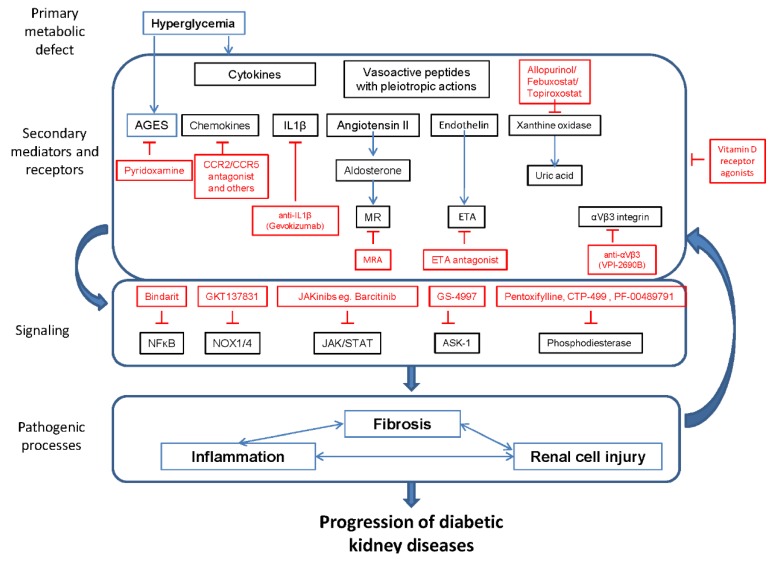
Promising therapeutic approaches to diabetic kidney disease (DKD) undergoing clinical trials. Only drugs and targets under active clinical investigation are shown. Renin angiotensin system (RAS) targeting drugs not shown as they are already in clinical use. MR: mineralocorticoid receptor. ETA: Endothelin receptor A.

The incidence and prevalence of diabetes mellitus (DM) is increasing worldwide due to evolving lifestyles and lower mortality from transmissible disease that is prolonging survival and increasing global aging [1]. There is a global trend towards a decrease in overall age-standardized mortality. Causes of death not following this trend deserve special attention [1]. In terms of ranking for years of life lost, chronic kidney disease (CKD) is the non-communicable cause of premature death that increased the most over the 1990–2013 period (90% increase), followed by diabetes (67% increase), according to the Global Burden of Disease 2013 study [1]. Both were among the top 25 causes of death, Much of the mortality due to CKD corresponded to diabetic kidney disease (DKD). Indeed, the age-standardized death rate from DKD increased by 106% in this period [1]. These data imply that the current approach to prevent and treat DKD, based on renin-angiotensin system (RAS) blockade and metabolic and blood pressure control should be further optimized to slow the growing epidemic [2,3]. Thus, a number of basic, translational and clinical research initiatives are aiming at improving the success rate of DKD therapy. Any new therapy should be more effective than angiotensin converting enzyme (ACE) inhibitors or angiotensin type 1 receptor blockers (ARBs) in preventing loss of renal function in head-to-head comparisons or improve outcomes when used in addition to RAS blockade. In this regard, the widespread use of RAS blockade may underlie the observation that while DKD is still the most frequent cause of incident end-stage renal disease (ESRD) (almost 50% according to 2014 United States Renal Data System (USRDS) data) [4], and among persons with diabetes, the prevalence of DKD remained stable (thus exposing patients with increased risk associated with CKD) [5]; it is also true that the number of incident cases has stabilized in recent years and the incidence rate has started a slow decrease and this may be related to wider implementation of nephroprotective strategies [6]. Ethics considerations limit the possibilities regarding head-to-head comparisons. Thus, most agents in the pipeline are being tested as add-on agents on top of RAS blockade. This is a rapidly evolving field, where some drugs are dismissed following early clinical trials, while new candidates appear in the horizon [7]. We now review the most likely candidates to be in the market by 2020, based on currently ongoing or recently completed clinical trials (Table 1, Table 2 and Figure 1).

**Table 1:** Therapeutic approaches to diabetic kidney disease (DKD) undergoing clinical trials with primary endpoint albuminuria or glomerular filtration rate. Most advanced clinical development phase is reflected [8].

Target	Drug	ClinicalTrials.gov Identifier	Phase	Enrollment (Expected)	Status	Completed Date (or Expectation)
Vitamin D receptor agonist	Paricalcitol	VITAL NCT00421733	II	281	Completed	June 2009
		PROCEED NCT01393808	II	112	Ongoing	June 2015
	Cholecalciferol	NCT00552409	II and III	22	Completed	June 2010
	Calcitriol	NCT01673204	IV	276	Ongoing	April 2014
Endothelin receptor antagonists	Avosentan	NCT00120328	III	2364	Terminated *	February 2007
	Atrasentan	NCT00920764	II	92	Completed	May 2010
		RADAR NCT01356849	II	149	Completed	August 2012
		SONAR NCT 01858532	III	4148	Ongoing	February 2017
Mineralocorticoid receptor blockers	Spironolactone	NCT00317954	IV	48	Completed	July 2005
		PRIORITY NCT02040441	II and III	3500	Ongoing	December 2017
	Eplerenone	NCT00315016	II	30	Completed	July 2011
	MT-3995	NCT01756703	II	67	Completed	August 2014
	BAY 94-8862 (Finerenone)	NCT01874431	II	821	Completed	August 2014
	Cs3150	NCT02345057	II	325	Ongoing	July 2016
Xanthine oxidase Inhibitor	Topiroxostat	NCT02327754	II	60	Ongoing	December 2016
	Allopurinol	PERL NCT02017171	III	480	Ongoing	December 2018
Phosphodiesterase inhibitor	Pentoxifylline	NCT00663949	II and III	70	Completed	January 2008
	PF0489791	NCT01200394	II	256	Completed	August 2013
	CTP499	NCT01487109	II	170	Completed	January 2015
Serotonin receptor antagonists	Sarpogrelate	SONATA NCT01869881	IV	166	Ongoing	December 2014
Nuclear factor erythroid 2-related factor 2 (Nrf2) activators	Bardoxolone RTA-402	NCT00811889	II	227	Completed	December 2010
		BEACON NCT00664027	III	2185	Terminated **	October 2012
		NCT02316821	II	72	Ongoing	December 2017
Chemokine inhibitors	PF-04634817	NCT01712061	II	226	Completed	September 2014
	CCX 140-B	NCT01447147	II	332	Completed	December 2014
	BMS-813160	NCT01752985	II	120	Ongoing	December 2015
NFκB inhibitors	Bindarit	NCT01109212	II	100	Completed	December 2008
Jakinibs	Baricitinib	NCT01683409	II	129	Completed	November 2014
Antioxidants	*N*-Acetylcysteine	NCT00556465	II and III	69	Completed	June 2007
		NCT00915200	II	225	Ongoing	March 2015
	NOX-E36	NCT01547897	II	76	Completed	December 2013
	Probucol	NCT01726816	II	126	Completed	September 2014
	Glutathione	NCT01265563	II	110	Ongoing	February 2015
	GKT137831	NCT02010242	II	200	Completed	March 2015
	Colchicine	NCT02035891	I, II, III and IV	160	Ongoing	June 2018
Galectin-3 antagonist	GCS-100	NCT02312050	II	375	Ongoing	Sep 2016
Integrin blocker	VPI-2690B	NCT02251067	II	300	Ongoing	August 2017
Apoptosis signal-regulating kinase 1	GS-4997	NCT02177786	II	300	Ongoing	August 2016
Antifibrotic therapies	Pirfenidone	NCT00063583	I and II	77	Completed	March 2009
	LY2382770 Anti-TGF-β1 mAb	NCT01113801	II	400	Completed ***	July 2014
RAGE inhibitor	TTP488	NCT00287183	II	110	Completed	August 2009
Glycosaminoglycans	Sulodexide	NCT00130208	III	1000	Completed	February 2008
		NCT00130312	IV	1248	Terminated ****	March 2008
		Soften NCT01316068	IV	80	Ongoing	August 2012
Metalloproteinase inhibitor	XL784	NCT00312780	II	125	Completed	December 2007
Inhibitors of epidermal growth factor ligands	TGF-α/epiregulin inhibitor LY3016859	NCT01774981	I and II	64	Ongoing	September 2015
ACTH receptor	ACTH	ACTH-NRDN NCT01028287	IV	15	Completed	July 2011
		NCT01601236	II	40	Ongoing	January 2015
DPP-4 Inhibitor	Linagliptin	NCT02376075	III	43	Completed	September 2014
		RENALIS NCT02106104	IV	48	Ongoing	May 2016
PKCβ inhibition	LY333531 (Ruboxistaurin)	NCT00044148	II	Not Provided	Completed	Not Provided
Inhibitors of AGE formation	Pyridoxamine	NCT00734253	II	317	Completed	August 2010
		PIONEER NCT02156843	III	600	Ongoing	December 2017

* Side effect safety concerns related to heart failure probably as a consequence of fluid retention; ** Independent Data Monitoring Committee recommendation for safety concerns; *** Although clinicaltrials.gov states “completed”, it was “terminated” for futility; **** No difference in protein excretion at 6 and 12 months. No safety issues; mAb, monoclonal antibody.

**Table 2:** Recently completed (from 2009) clinical trials enrolling >50 patients with results available.

Drug	Comparator	Months	*N*	Study Population	Primary Endpoint	Results
Paricalcitol	Placebo *vs.*(low and high dose of drug)	6	281	T2DM, on RAS blockade. GFR: 15–90. UACR: 100–3000	UACR	Low dose group: −14%, High dose group: −20, Placebo group: −3%. Drug *versus* placebo −15% (*p* = 0.071)
Atrasentan	Placebo (*vs.* 3 doses of the drug)	2	89	T2DM, on RAS blockade. GFR: >20 UACR: 100–3000	UACR	Reduction by 35%–42% *versus* 11% for placebo (*p* < 0.005)
	Placebo (*vs.* 2 doses of the drug)	3	211	T2DM, on RAS blockade. GFR: 30–75 UACR: 300–3500	UACR	Reduction by 35%–38%
BAY 94-8862 (Finerenone)	Placebo (*vs.* 7 doses of the drug)	3	821	T2DM, on RAS blockade. GFR: 30–90 UACR: 30–300 and 300–3000	UACR	Dose-dependently reduced UACR. Mean ratio of UACR in the two highest doses *vs.* placebo was 0.62 and 0.67 (*p* < 0.0001 either)
PF0489791	Placebo	3	256	T2DM, on RAS blockade. GFR: 30–90 UACR: >300	UACR	Significant reduction in UACR (15.7%) compared to placebo
CTP499	Placebo	12	177	T2DM, on RAS blockade. GFR: 23–89. UACR: 200–5000 if male 300–5000 if female	UACR after 24 weeks	Failed to meet the primary endpoint. Serum creatinine after 48 weeks lower (mean increase in CTP499; 0.13 mg/dL *versus* Placebo: 0.21 mg/dL, *p* = 0.057)
Bardoxolone RTA-402	Placebo (*vs.* 3 doses of the drug)	12	227	T2DM, on RAS blockade. GFR: 20–45	GFR at 24 weeks	Significant increases in GFR, as compared with placebo (low dose group: +8. Medium dose: +11. High dose: +10 (*p* < 0.001).
CCX 140-B	Placebo	13	332	T2DM, on RAS blockade. GFR: >25 UACR: 100–3000	UACR	Decreased albuminuria by 24% and after an initial reduction in eGFR, decreased the slope of eGFR loss
Pirfenidone	Placebo (*vs.* 2 doses of the drug)	12	77	DMT1 and T2DM, not specifically on RAS blockade. GFR: 20–75	GFR after 1 year	Mean GFR increased in pirfenidone +3.3 whereas decreased in placebo −2.2 (*p* = 0.026)
LY2382770 Anti-TGF-β1 mAb	Placebo	12	416	DMT1 and T2DM, on RAS blockade. GFR: PCR > or equal 800	Serum creatinine	Terminate: futility
Pyridoxamine	Placebo (*vs.* 2 doses of the drug)	12	317	T2DM, on RAS blockade. sCr 1.3–3.3 female or 1.5–3.5 male. PCR > 1200	Serum creatinine	Failed to meet primary endpoint. Subgroup analysis: in the lowest tertile of baseline sCr, Pyridorin associated with a lower average change in serum creatinine concentration at 52 weeks (drug 1: −0.28 drug 2: 0.07 placebo: 0.14 (*p* = 0.05)

T2DM: type 2 diabetes mellitus; eGFR: estimated glomerular filtration rate in mL/min/1.73 m^2^; sCr: serum creatinine in mg/dL; UACR: urinary albumin-to-creatinine ratio in mg/g; P24h: proteinuria g/24 h; PCR: protein/creatinine ratio in mg/g; mAb, monoclonal antibody.

## 2. Current Therapy for Diabetic Kidney Disease

DKD is characterized by increasing albuminuria that progresses from A1 category of the 2012 KDIGO classification of CKD (urinary albumin/creatinine ratio (UACR) 30–300 mg/g) to A2 (UACR 300 mg/g) and is followed by a gradual decrease in glomerular filtration rate (GFR), leading to end-stage renal disease (ESRD) [9]. Residual albuminuria after initiation of RAS blockade is the main risk factor for progression of DKD. However, GFR may be reduced in the absence of significant albuminuria in type 2 DM (T2DM) [10,12,13]. Non-proteinuric DKD usually progresses more slowly. Hyperuricemia and systemic inflammation are risk factors for progression. Direct induction of tubular cell stress by high glucose levels and glucose degradation products may elicit pro-inflammatory and fibrogenic response even when albuminuria is low [14,15]. ACE inhibitors or ARBs control blood pressure, reduce proteinuria and slow the loss of renal function in patients with DKD and UACR >30 mg/g [3] or hypertension and normoalbuminuria [16]. Dual RAS blockade (ACE inhibitor plus ARB) is not recommended by Kidney Disease Outcome Quality Initiative. (KDOQI), Kidney Disease Improving Global Outcomes (KDIGO) or American Diabetes Association (ADA) guidelines as no additional efficacy in terms of renal function has been demonstrated and the incidence and severity of adverse effects is increased [3,17,18,19]. However, some experts disagree with this recommendation [20,21].

The number of promising drugs that failed at the clinical trial stage in the field of DKD is ever increasing. Most clinical trials had as primary outcomes albuminuria or eGFR, and the interventional drug was usually added to RAS blockade. Either lack of efficacy or safety concerns were the reasons for failure. Bardoxolone methyl, inhibitors of protein kinase C (ruboxistaurin) or of AGEs formation (pimagedine/aminoguanidine, pyridoxamine), and sulodexide, have failed to find a place in the treatment of DKD [7,22]. Pirfenidone, anti-connective tissue growth factor (CTGF) or, anti-TGF-β monoclonal antibodies, and antifibrotic therapies are no longer being pursued in patients with DKD [7]. The early termination of a phase 2 clinical trial of the neutralizing anti-TGF-β1 antibody LY2382770 due of futility to preserve renal function in DKD was announced at the 2014 American Society of Nephrology meeting [23]. Pyridoxamine is still being pursued in an ongoing phase 3 trial based on *post-hoc* analysis of phase 2 data that suggested a non-dose-dependent benefit in serum creatinine with marginal statistical significance [23,24].

## 3. Ongoing Clinical Trials

Currently ongoing randomized controlled trials in DKD are further exploring tested concepts (as an example, endothelin receptor antagonism with improved molecules or vitamin D receptor activation), old drugs (e.g., allopurinol) or novel therapeutic approaches (e.g., targeting fibrosis or inflammation). Inflammation is considered a key contributor to progression of DKD and positive results were recently reported for anti-inflammatory agents in clinical trials [25]. Of ongoing trials, only the phase 3 atrasentan RCT may result in a new therapeutic indication, while most other trials will provide proof-of-concept. Most clinical trials of nephroprotective agents in DKD use albuminuria as the primary outcome because this design enables a shorter follow-up duration and smaller sample size [26,27,28,29,30,31]. However, these are usually phase 2 data that require demonstration in phase 3 studies that GFR is also preserved. In these regard, trials assessing GFR and specially measured GFR are more relevant. Recently, a 30% reduction in eGFR over two years was reported to be a more frequent outcome than doubling of serum creatinine and to be strongly associated with the risk of ESRD [32]. Thus, this endpoint may be considered as an end point for CKD progression, especially for drugs with no hemodynamic actions.

### 3.1. Optimizing Already Tested Approaches or Drugs

Some ongoing trials are exploring drugs targeting molecular mechanisms that have already been successfully targeted for kidney injury or other diseases.

#### 3.1.1. Vitamin D Receptor Activators

Vitamin D receptor (VDR) activation has anti-inflammatory, immunologic and nephroprotective actions [33]. Activation of podocyte VDR protects from inflammation or fibrosis triggered by metabolic abnormalities [34,35]. Diabetic animals that lack VDR develop albuminuria, whilst VDR activation by paricalcitol (19-nor-1,25-(OH)_2_-vitamin D_2_) or calcitriol decreases proteinuria [33,34]. In small cohorts paricalcitol decreased proteinuria [36,37,38]. However, a phase 2 RCT (VITAL) exploring the antiproteinuric effect of 1 μg or 2 μg/24 h paricalcitol as add-on to RAS blockade in CKD stages 2–4 DKD failed to meet the primary end-point (change in UACR at 24 weeks: group difference for paricalcitol *versus* placebo of −15%, *p* = 0.071) [26]. *Post-hoc* analysis disclosed that the higher dose decreased albuminuria in patients with high salt ingestion. The study was marred by the high prevalence of vitamin D deficiency that did not allow discrimination of therapeutic effects of paricalcitol from replacement of vitamin D [39] and was probably underpowered. The Antiproteinuric Effect of Selective Vitamin d Receptor Activation by Paricalcitol in Type 2 Diabetes Patients on Low or High Sodium Diet and Stable Ras Inhibitor Therapy (PROCEED) trial [23] is exploring the antialbuminuric effect of 2 μg/24 h paricalcitol or placebo for four months on top of RAS blockade in patients with T2DM and UACR >300 mg/24 h who have low or high salt intakes, in order to clarify the interaction between salt intake and VDR activators.

#### 3.1.2. Endothelin Receptor Antagonists

Endothelins are small pleiotropic peptides that promote hypertension, albuminuria, insulin resistance, inflammation, fibrosis and endothelial dysfunction [27]. Endothelin-1 activates receptors ETA, ETB1 and ETB2 [27]. ETA activation is thought to have disease-causing effects, whereas ETBs are natriuretic [27,40,41,42]. However, even very selective ETA blockers cause fluid retention. This side effect led to the termination of a phase 3 trial of avosentan in DKD, despite a significant reduction in albuminuria (44%–49% as compared with 10% for placebo) when added to RAS blockade [43].

Atrasentan is a more selective ETA antagonist than avosentan that in phase 2 trials reduced UACR by 35%–42% *versus* 11% for placebo in DKD. Unfortunately, atrasentan caused dose-dependent peripheral edema (18%–46% of patients) [27]. A larger phase 2 trial (RADAR) confirmed a reduction of albuminuria of 35%–38%, but found no differences in peripheral edema, although atrasentan patients gained more weight and hematocrit was lower, suggesting volume overload [44]. For this reason the currently ongoing SONAR phase 3 trial has strict entry criteria that exclude patients at risk of fluid overload. This may be a major limitation to the wider use of the drug if it becomes commercially available. SONAR is assessing the effect of atrasentan *versus* placebo as add-on to RAS blockade in 4148 patients with T2DM, eGFR 25–75 mL/min/1.73 m^2^ and UACR 300–5000 mg/g [23]. The primary outcome is time to the first occurrence of a composite renal end point (doubling of sCr levels or development of ESRD). SONAR is expected to be completed by February 2017.

#### 3.1.3. Mineralocorticoid Receptor Antagonists

Aldosterone secreted in response to RAS activation binds and activates the mineralocorticoid receptor to regulate sodium balance and to promote inflammation and fibrosis [45]. Mineralcorticoid receptor antagonists as add-on to RAS blockade decrease albuminuria in diabetic and non-diabetic kidney disease, but their effect on long-term renal function has not been explored and may increase the risk of hyperkalemia [21,46,47]. The PRIORITY clinical trial is investigating the efficacy of the non-expensive, first generation mineralocorticoid receptor antagonist spironolactone for prevention of albuminuria in T2DM patients with normoalbuminuria at high risk of progression based on urinary proteomics assessment [48,49].

Completed phase 2 trials that have tested the effect on albuminuria of newer, selective, nonsteroidal mineralocorticoid receptor antagonist MT-3995 or finerenone (BAY 94-8862) reported to have lower risk of hyperkalemia on top of RAS blockade in DKD (eGFR 30–60 mL/min/1.73 m^2^ and albuminuria ≥300 mg/g) [46,50]. An abstract reported that finerenone was safe and decreased UACR and blood pressure at three months [51] and the drug is expected to enter phase 3 development in 2015.

#### 3.1.4. Xanthine Oxidase Inhibitors

High serum uric acid levels are associated with the development and progression of DKD [52] and an open label clinical trial suggested that allopurinol may provide nephroprotection [53]. Ongoing clinical trials are addressing nephroprotection by commercially available xanthine oxidase inhibitors allopurinol and febuxostat. PERL is a phase 3 trial assessing the impact of allopurinol on eGFR in 480 patients with T1DM, albuminuria and CKD stage 1 to 3A [54]. Nephroprotection by febuxostat is addressed by the ongoing FEATHER trial, which has as primary endpoint eGFR in patients with stage 3 CKD and hyperuricemia but is not limited to diabetics [55]. The phase 2 study UPWARD is assessing topiroxostat effects on albuminuria in diabetic patients with hyperuricemia or gout and will be completed in December 2016 [23].

#### 3.1.5. Phosphodiesterase Inhibitors

Pentoxifylline is an anti-inflammatory phosphodiesterase inhibitor, which is used primarily to treat peripheral vascular disease. A randomized controlled trial reported that adding low-dose pentoxifylline (400 mg daily) to dual RAS blockade (losartan plus enalapril) in 50 patients with T2DM resulted in a decrease in urinary protein excretion from 616 mg/day at baseline to 192 mg/day at 6 months (*p* = 0.000) [56]. The Pentoxifylline for Renoprotection in Diabetic Nephropathy (PREDIAN) study was an independent, open-label, randomized controlled clinical trial that explored add-on pentoxifylline to maximized RAS blockade on renal disease progression in T2DM patients with CKD stages 3–4. PREDIAN randomized 169 Caucasians patients (mean age 70 years, mean duration of DM 15 years) to a control group or a treatment group that received pentoxifylline (1200 mg/day). The mean baseline eGFR was 37 ± 12 mL/min/1.73 m^2^, 69% of patients had GFR category G3 and 91% had UACR >300 mg/g. All patients were hypertensive, most had hyperlipidemia, and more than 40% had medical history of coronary heart disease. After 24 months of follow-up, treatment with pentoxifylline was associated with a slower rate of eGFR loss: in control patients eGFR was 4.3 mL/min/1.73 m^2^ lower than in pentoxifylline-treated patients. The difference in the reduction of eGFR between the groups reached statistical significance after one year, suggesting that a long-period of pentoxifylline treatment is necessary to achieve a protective effect on renal function. In addition, this study confirmed the additive antiproteinuric effect of pentoxifylline in T2DM with residual proteinuria, with a mean difference of 21% in favor or pentoxifylline respect to the control group, an effect that was significant from the sixth month [57].

CTP-499 incorporates deuterium at select positions of 1-((*S*)-5-hydroxyhexyl)-3,7-dimethylxanthine (HDX), an active metabolite of pentoxifylline, improving the metabolism profile [58]. Results from a phase 2 trial reported in abstract form indicate that in 182 patients with T2DM DKD (UACR 300–5000 mg/g; eGFR 23–89 mL/min/1.73 m^2^) 600 mg PO BID CTP-499 failed to meet the primary endpoint (change in UACR after 24 weeks), but a promising effect on serum creatinine was observed after 48 weeks (mean increase in serum creatinine levels 0.13 mg/dL *versus* 0.21 mg/dL, *p* = 0.057). Furthermore, only 1.5% (1/65) of patients in the CTP-499 group experienced a ≥50% increase in sCr, *versus* 5/58 (8.6%) of patients on placebo (*p* < 0.05). The rise in UACR was lower in patients receiving CTP-499 (24 mg/g *versus* 222 mg/g on placebo, *p* = 0.13) [59]. The company announced in July 2014 that it would pursue phase 3 trials, but no new trials were posted at clinicaltrials.gov as of 1 April 2015.

PF-00489791 is expected to have mainly hemodynamic actions by increasing the intracellular cGMP pool through inhibition of the cGMP hydrolyzing enzyme phosphodiesterase type 5. A recently completed 12-week phase 2 clinical trial in DKD reported in abstract form a significant reduction in UACR (16%) [60].

#### 3.1.6. Serotonin Receptor Antagonists

Sarpogrelate is a 5HT2A/5-HT2B receptor antagonist antiplatelet agent that is as effective as aspirin in preventing macrovascular complications of T2DM and is commercially available in several countries [61]. Sarpogrelate decreased type IV collagen production by cultured mesangial cells [62] reduced albuminuria is experimental DKD, [63] and decreased antibody-mediated glomerular injury [64] and nephrotoxin-induced kidney fibrosis, suggesting mechanisms of action beyond the effects on platelets [65]. The primary outcome of an ongoing phase 4 trial (SONATA) is UACR in T2DM.

### 3.2. Novel Therapeutic Approaches

Some trials are exploring or have recently explored novel therapeutic targets or drugs in DKD.

#### 3.2.1. Nuclear Factor Erythroid 2-Related Factor 2 (Nrf2) Activators

Bardoxolone methyl is a synthetic triterpenoid activator of the transcription factor Nrf2 and an inhibitor of nuclear factor κB (NFκB) [66]. In a promising phase 2 RCT bardoxolone methyl increased estimated GFR in DKD at the expense of higher blood pressure and albuminuria [29]. In addition, anorexia, weight loss and hypomagnesemia were noted. A phase 3 trial (BEACON) confirmed these actions and side effects, but was terminated for safety concerns related to heart failure probably as a consequence of fluid retention [67]. This was probably the end of the road for bardoxolone methyl in DKD. Signs of toxicity had already been reported in experimental CKD, although they were dismissed as a consequence of a defective batch of the drug [68]. Nevertheless, Nrf2 activators such as dimethyl fumarate are currently used to treat multiple sclerosis [69,70] and new trials are exploring the safety and tolerability of the bardoxolone methyl formulation RTA 402 [23]. Thus, Nrf2 activators for DKD may not be entirely dismissed. Either successful and safe use for another indication or observations of improved kidney outcomes in diabetic multiple sclerosis patients may trigger a second round of interest in bardoxolone methyl or other Nrf2 activators for DKD. Indeed, despite worldwide termination of bardoxolone methyl clinical research programs for DKD following the termination of the BEACON trial, a reanalysis of the potential risk/benefit of the drug for the Japanese population led to reinitiating RCTs for DKD in Japan in 2015. Since the incidence of cardiovascular events is lower in Japanese CKD patients with DM than in European or US patients, it was hypothesized that a dose-escalation study in this population may be devoid of the risks observed in BEACON; in which most of the severe adverse effects were concentrated within the first month of therapy. A placebo-controlled RTA 402 Phase 2 Clinical Trial (A Randomized, Double-blind, Placebo-controlled Clinical Trial in Patients with Chronic Kidney Disease and Type 2 Diabetes, TSUBAKI, NCT02316821) is currently recruiting participants in Japan. The purpose is to assess the safety and efficacy (insulin clearance) of RTA 402 in 72 CKD patients with T2DM when administered once daily for 16 weeks in an intrapatient dose escalation design. The estimated study completion date is December 2017. Dose and inclusion criteria were not available at clincialtrials.gov. However, several issues remain unsolved: the rapid time-course of the increase in estimated GFR suggests a hemodynamic basis. If this was the case, then bardoxolone methyl actions would be the complete opposite to the current standard of therapy (RAS blockade): bardoxolone methyl appears to increase GFR, albuminuria and blood pressure through a hemodynamic effect, while RAS blockade decreases these three parameters and it is precisely the decrease of the three parameters that is thought to contribute to nephroprotection. As a consequence, if bardoxolone methyl were indeed successful at preventing CKD progression, the whole concept of nephroprotection in DKD would need to be rewritten.

#### 3.2.2. Chemokine Inhibitors

High glucose elicits a pro-inflammatory response in tubular cells and podocytes characterized by chemokine secretion that promotes kidney inflammation [71]. An ongoing phase 2 trial is exploring the safety, tolerability and effect on albuminuria of the chemokine receptor BMS-813160. A phase 2 trial of CCR 2/5 antagonists PF-04634817 was recently completed but results are not yet available. Another recently completed phase 2 trial provided promising results for CCX140, an oral small molecule inhibitor of CCR2. Targeting the chemokine MCP-1/CCL2 or its receptor CCR2 by different means (neutralizing antibodies, receptor antagonists, inhibitors, DNA vaccines, mutant genes or enantiomeric RNA oligonucleotides (l-RNA aptamer )) decreased albuminuria, kidney injury and inflammation and preserved kidney function in experimental kidney injury, including DKD [71,72,73]. CCX140 improved metabolic control in diabetic mice [74] and decreased albuminuria and podocyte injury and improved glycemic control in transgenic diabetic human CCR2 knockin mice [73]. A press report indicated that CCX140 improved the metabolic profile in human T2DM in phase 2 trials. Furthermore, over 52 months, 5 mg/day CCX140 decreased albuminuria by 24% and after an initial reduction in eGFR, decreased the slope of eGFR loss [75]. While the data remain unpublished, phase 3 trials are being designed.

#### 3.2.3. Anti-IL-1β Antibodies

Gevokizumab is a potent recombinant monoclonal antibody, with unique allosteric modulating properties [76]. It binds to human interleukin-1 beta (IL-1β), a pro-inflammatory cytokine, with high affinity resulting in inhibition of IL-1β signaling and pro-inflammatory activity [77]. Gevokizumab is currently being studied in multiple diseases, including global Phase 3 clinical programs in chronic inflammatory disease such as Behçet’s disease, uveitis, non-infectious uveitis, and pyoderma gangrenosum [78]. A dose-response study of gevokizumab in patients with T2DM and DKD with a primary outcome of measured GFR will start in 2015 [79].

#### 3.2.4. NFκB Inhibitors

NFκB is a key contributor to experimental kidney injury and there is evidence for NFκB activation in human DKD [80]. Bindarit (2-((1-benzyl-indazol-3-yl) methoxy)-2-methyl propionic acid) downregulates the activation of specific NFκB dimers, reducing chemokine expression [81]. In lupus nephritis phase 2 trials, bindarit for 24 weeks reduced albuminuria and urinary CCL2 excretion [82]. A small phase 2 trial in 100 patients with DKD (NCT 01109212) reported that bindarit administration for 12 weeks reduced albuminuria, [83] but the full results have not been published and no new trials of this agent are ongoing.

#### 3.2.5. Jakinibs

Jakinibs are small molecule inhibitors of Janus kinases (JAK) activation. JAKs activate the family of transcription factors of Signal Transducer and Activator of Transcription (STAT) which are thought to promote hyperglycemia-induced kidney injury. Dysregulation of the JAK-STAT pathway has been documented in human progressive DKD [84]. Inhibitors of JAK2 (AG-490) which might have additional cytoprotective effects such as activation of hypoxia inducible factor 1 (HIF-1**)** [85,86]. JAK3 (Janex-1) and STAT1 (fludarabine) have been proposed as anti-diabetic agents [87,88]. Overexpression of suppressors of cytokine signaling (SOCS)-1 and SOCS-3, the intracellular negative regulators of JAK-STAT signaling is also protective in experimental DKD [89]. The oral JAK1 and JAK2 inhibitor baricitinib (formerly LY3009104, INCB28050), which is in phase 3 development for rheumatoid arthritis, [90] might also have renoprotective effects. A recently completed phase 2 trial tested baricitinib or placebo as add-on to RAS blockade in 129 patients with DKD and UACR 300–5000 mg/g. The primary outcome is change from baseline UACR at 24 weeks of treatment. Study results are still pending publication [23].

#### 3.2.6. Antioxidants

Oxidative stress is a contributor to tissue injury in DKD and other diseases, but has proved elusive as a therapeutic target. GKT137831 is a NOX1/4 inhibitor undergoing a phase 2 trial that was completed in February 2015, but results are not available yet [23].

#### 3.2.7. Galectin-3 Antagonist

Galectin-3 is a lectin that regulates cell proliferation, apoptosis, cell adhesion and affinity for advanced glycation end products and is upregulated in experimental and human DKD and ESRD [91,92,93,94,95]. In diabetic patients, glomerular galectin-3-positive cells correlated with decreasing renal function [96]. In human cohorts, galectin-3 levels increased with progressive renal impairment and were independently associated with all-cause death [97]. A clinical trial is exploring the effect of the galectin-3 antagonist GCS-100 for 26 weeks on eGFR in DKD [23]. However, some data point to a requirement of galectin-3 for nephroprotection. Thus, galectin-3 knock-out mice develop more severe DKD as well as spontaneous age-dependent glomerular lesions [98,99].

#### 3.2.8. Integrin Blocker

VPI-2690B is an antibody against the αVß3 integrin receptor (vitronectin receptor) that is currently undergoing phase 2 RCTs in DKD with decrease of albuminuria as primary outcome [23]. αVβ3 allows interaction of the podocyte with matrix proteins [100]. Expression of αVβ3 receptor and its ligand vitronectrin is increased in experimental DKD [101]. αVβ3 integrin receptor activation is thought to contribute to podocyte injury when activated by soluble urokinase plasminogen activator receptor (suPAR) in the presence of low podocyte-specific acid sphingomyelinase-like phosphodiesterase 3b (SMPDL3b) levels. However, in DKD both serum suPAR and glomerular SMPDL3b expression are increased. Under these conditions, SMPDL3bwas reported to prevent αVβ3 integrin activation by suPAR and this resulted in podocyte cell death in cultured cells [102]. While the pathogenicity of suPAR in DKD in debated, these observations raise concerns over the safety of VPI-2690B, since by preventing αVβ3 in a high suPAR context it may favor podocyte death. However, VPI-2690B was observed to decrease proteinuria, diminish glomerular basement membrane thickening and podocyte foot process effacement in experimental DKD in pigs [103].

#### 3.2.9. Apoptosis Signal-Regulating Kinase 1 (ASK1, Mitogen-Activated Protein Kinase Kinase Kinase 5, MAP3K5) Inhibitors

ASK-1 is a member of the MAP kinase kinase kinase superfamily that activates c-Jun *N*-terminal kinase (JNK) and p38MAPK in a Raf-independent fashion in response to oxidative stress and inhibited by the endogenous molecule thioredoxin [104,105]. ASK-1 targeting protected from diabetes-induced endothelial dysfunction, endothelial cell senescence and endoplasmic reticulum stress in experimental DKD or cultured cells [104,106,107]. ASK1 may also be diabetogenic [108]. The ASK1 inhibitor GS-4997 is undergoing RCT in DKD with albuminuria and estimated GFR as primary outcome [23].

## 4. Conclusions

Treatment of DKD still relies on RAS targeting drugs, which were first observed to reduce albuminuria over 30 years ago. The lack of advances in this area is worrisome, given the increase in global premature mortality from CKD and DKD in recent decades. While adequate blood pressure control, low salt diet and adequately dosed single RAS blockade may slow or stabilize the rate of progression of DKD in some patients, DKD still remains the most frequent cause of ESRD. Despite several setbacks, the field of clinical trials for add-on therapies for DKD on top of RAS blockade is currently active. Recent success with pentoxifylline on renal function preservation, positive preliminary data with chemokine targeting drugs and successful phase 2 trials with endothelin receptor antagonists point towards inflammation as the common denominator underlying promising therapeutic approaches. Ongoing phase 3 trials with atrasentan and soon to start trials with gevokizumab provide the basis for cautious optimism for the future.
